# Lateral open wedge calcaneus osteotomy with bony allograft augmentation in adult acquired flatfoot deformity. Clinical and radiological results

**DOI:** 10.1007/s00590-021-02888-3

**Published:** 2021-02-12

**Authors:** Gabriele Colo’, Mattia Alessio Mazzola, Giulio Pilone, Giacomo Dagnino, Lamberto Felli

**Affiliations:** 1 Orthopaedic Clinic, Ospedale Policlinico San Martino, Largo Rosanna Benzi 10, 16132 Genova, Italy; 2 Department of Surgical Sciences (DISC), Università degli Studi di Genova, Viale Benedetto XV, 6, 16132 Genova, Italy

**Keywords:** Open wedge, Flat foot, Calcaneus osteotomy, Posterior tibialis tendon, Gait analysis

## Abstract

**Abstract:**

The aim of this study is to evaluate the results of patients underwent lateral open wedge calcaneus osteotomy with bony allograft augmentation combined with tibialis posterior and tibialis anterior tenodesis. Twenty-two patients underwent adult-acquired flatfoot deformity were retrospectively evaluated with a minimum 2-year follow-up. Radiographic preoperative and final comparison of tibio-calcaneal angle, talo–first metatarsal and calcaneal pitch angles have been performed. The Visual Analog Scale, American Orthopedic Foot and Ankle Score, the Foot and Ankle Disability Index and the Foot and Ankle Ability Measure were used for subjective and functional assessment. The instrumental range of motion has been also assessed at latest follow-up evaluation and compared with preoperative value. There was a significant improvement of final mean values of clinical scores (*p* < 0.001). Nineteen out of 22 (86.4%) patients resulted very satisfied or satisfied for the clinical result. There was a significant improvement of the radiographic parameters (*p* < 0.001). There were no differences between preoperative and final values of range of motion. One failure occurred 7 years after surgery. Adult-acquired flatfoot deformity correction demonstrated good mid-term results and low recurrence and complications rate.

**Level of evidence:**

Level 4, retrospective case series.

## Introduction

Adult-acquired flat foot deformity (AAFD) represents a common debilitating pathology that leads to collapse of the medial longitudinal arch of the foot [1].

The tibialis posterior tendon (TPT) insufficiency represents the most common cause of AAFD but several other causes are described in the literature (rheumatoid arthritis, trauma, secondary Charcot foot, peripheral neuropathies and tumors) [2–4].

The spring ligament, the anterior fibers of deltoid ligament, the plantar fascia, the plantar and talo-calcaneal interosseous ligaments, and the talo-navicular (TN) and naviculo-cuneiform joints sustain the medial longitudinal arch [4]. The typical pes planovalgus deformity of AAFD is caused by progressive failure of this osteo-ligamentous complex [4].

Several operative approaches have been described for the different stages of foot involvement [5–7].

The osteotomy of calcaneus is widely described in the literature with encouraging results [8–11], but the importance of the soft tissues and TN joint restoration is often understated [2, 11].

Only one article reported the long-term results of lateral open wedge calcaneus osteotomy technique combined with tenodesis of TPT and tibialis anterior tendon (TAT) [11] and, for the best of our knowledge, no one reported the clinical and radiographical results in patients underwent calcaneal osteotomy combined with TPT and TAT tenodesis.

The aim of this study is to evaluate the clinical and radiological mid-term outcome and the instrumental range of motion (ROM) of patients underwent correction of AAFD with lateral osteotomy of the calcaneus and bony allograft augmentation combined with TPT and TAT tenodesis with a minimum 2-year follow-up.

## Materials and methods

This study obtained the Institutional Review Board approval and written and informed consent was obtained from each patient prior to study inclusion.

Clinical data and radiographs of patients treated with open wedge calcaneus osteotomy for AAFD at our Institution from January 2008 to December 2015 were retrieved and reviewed.

Inclusion criteria were: patients affect by AAFD with TPT insufficiency (stage II), unresponsive to conservative measures (non steroidal anti-inflammatory drugs, orthotics and physical therapy) for at least 6 months [2].

Exclusion criteria were: associated TN arthrodesis, history of diabetes, less than 2-year of follow-up and bilateral surgery.

All patients were preoperatively classified with four grades of Myerson classification [4].

All patients presented the “too many toes” sign [2], inability to perform a single heel rise, pain with swelling along TPT and mobile mid-foot and hind-foot. The TPT pathology was preoperatively confirmed with high-resolution ultrasound (US) or magnetic resonance imaging (MRI) in all cases. Severe abnormal changes of TPT or complete tendon rupture at preoperative imaging studies represented an absolute contraindication for tenodesis.

The measurements of the foot alignment were obtained with digital measurement by single independent observer (G.C.) from preoperative and final weight-bearing X-rays. The Saltzman view [12] was used to assess the hindfoot alignment. The tibio-calcaneal angle (TCA) was obtained between the weight-bearing axis of the tibia and the long axis of the calcaneus from the heel contact point [13].

On the lateral weight-bearing view the talo–first metatarsal (LTMT) and calcaneal pitch (CP) angles were measured [13, 14].

**Fig. 1:**
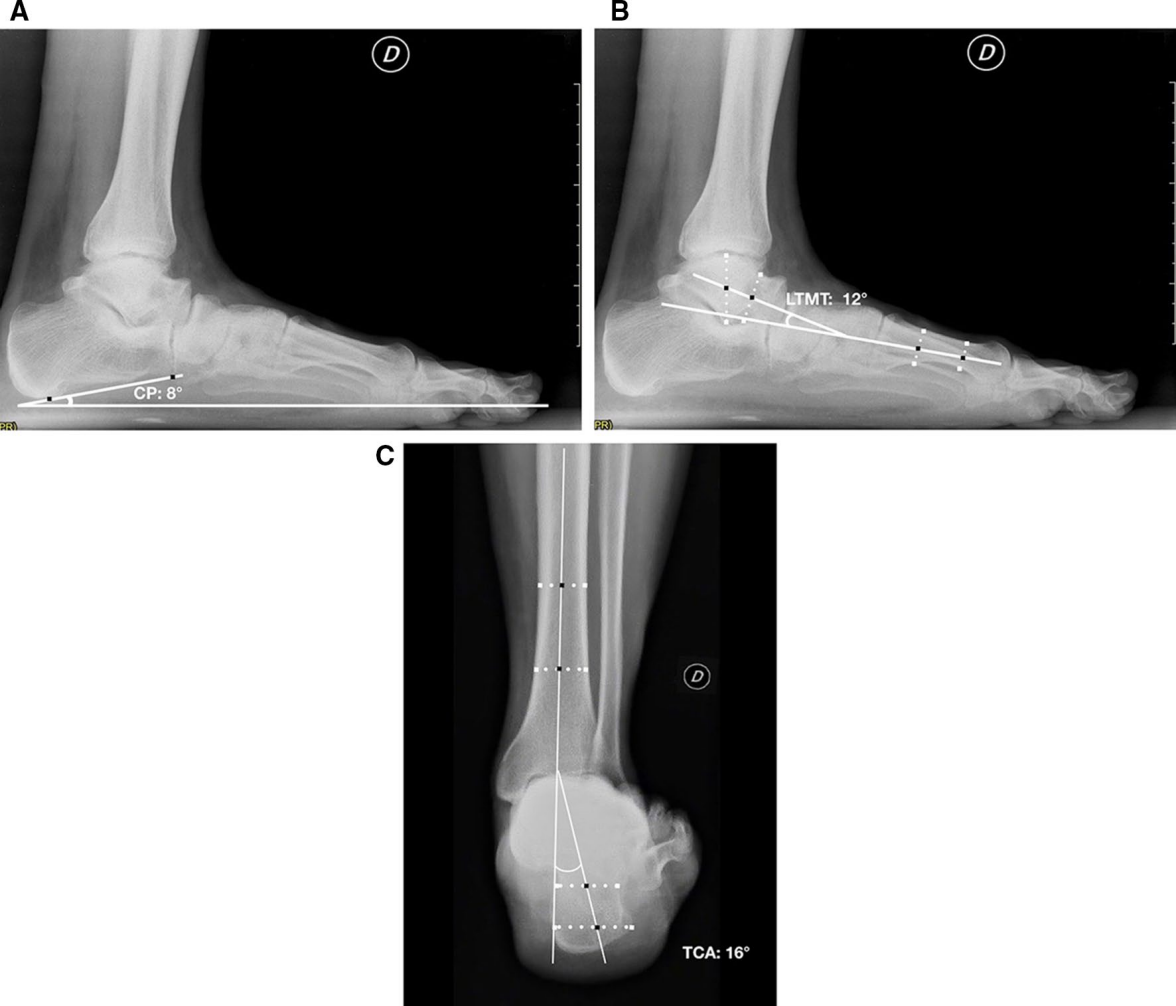
preoperative weight-bearing lateral view of a 68-year-old male patient with AAFD. The X rays show the assessment of calcaneal pitch (CP) (**a**) and talo–first metatarsal (LTMT) angles (**b**). The Saltzman view **c** shows the tibio-calcaneal angle (TCA) as a measurement of hindfoot alignment

**Fig. 2:**
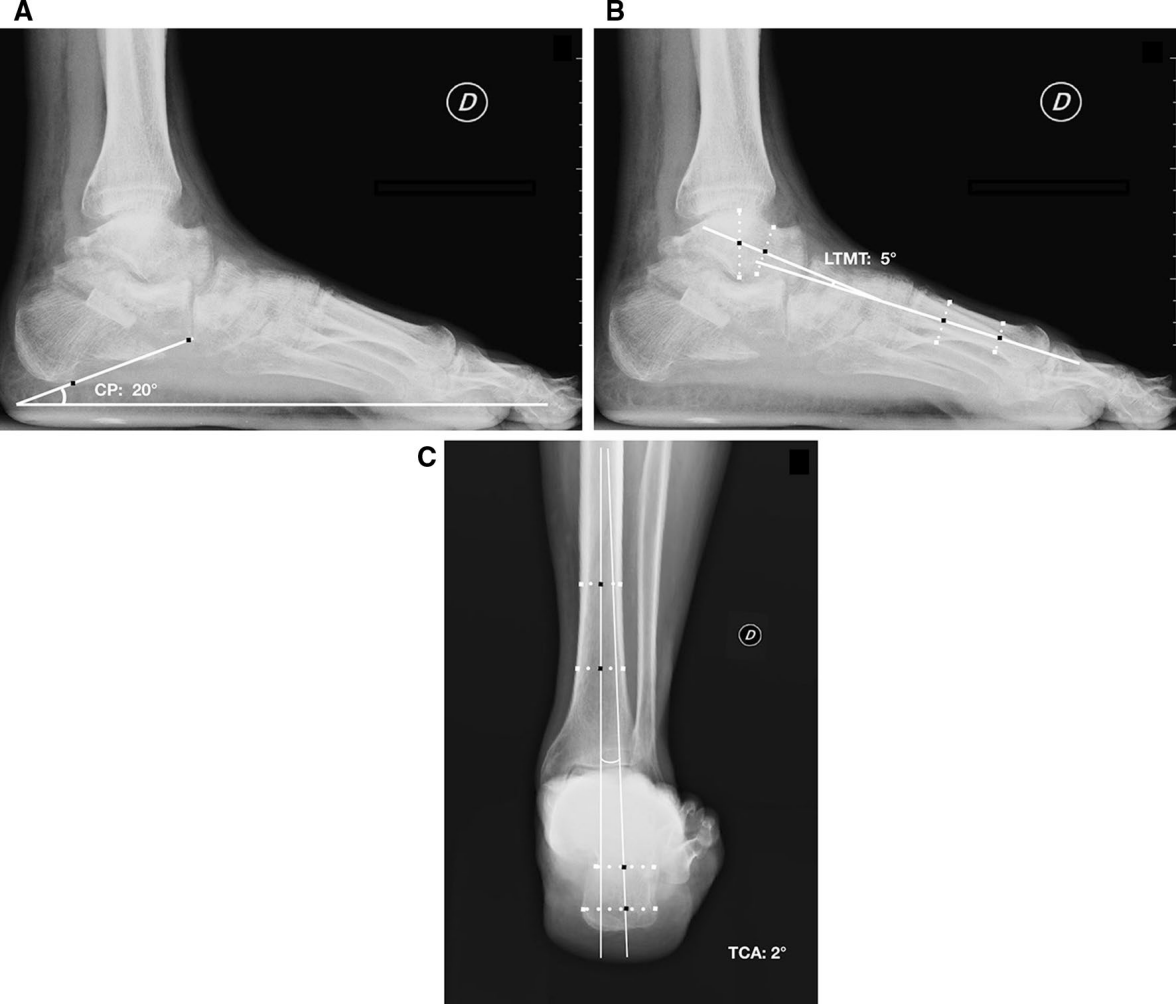
Weight-bearing lateral view of the same patient 3 months after calcaneal osteotomy with allograft augmentation and TAT and TPT tenodesis. The improvement of calcaneal pitch (CP) (**a**) and talo–first metatarsal angles **b** is measured and the Saltzman view **c** shows the reduction of tibio-calcaneal angle (TCA)

Figures 1 and 2 show an example of radiographic examination of the foot and the measurement of radiographic parameters.

All included patients were clinically reassessed by a single board-certified foot surgeon (D.G.) at the end of follow-up.

Preoperative data were collected with the inspection of all medical records and patients were recalled to respond to different surveys concerning the postoperative level of joint function and subjective level of pain.

At the end of the follow-up the level of satisfaction and the visual analog score (VAS) was assessed to report the subjective grading of pain.

The American Orthopedic Foot and Ankle Score (AOFAS), the Foot and Ankle Disability Index (FADI) and the Foot and Ankle Ability Measure (FAAM) were used for functional evaluation and preoperative and postoperative pain, recorded on the central hospital database.

The included patients were clinically evaluated at the end of follow-up and patients who refused to participate to final clinical assessment were excluded.

The F4A sensors (Free4Act^®^—F4A, LorAn Engineering) have been used to conduct the accelerometric analysis of the active articular ROM (plantar flexion, dorsal flexion, pronation and supination) investigating the impaired joint. preoperative data of ROM were compared to final values.

Failure was defined as recurrence or revision surgery (any other surgery at the affected foot). Reported complications (complex regional pain syndrome (CRPS), infection, wound leakage, neurovascular damage) have been documented and analyzed as well.

All surgeries were performed with spinal anesthesia, supine position and with the use of the tourniquet (250 mmHg).

**Fig. 3:**
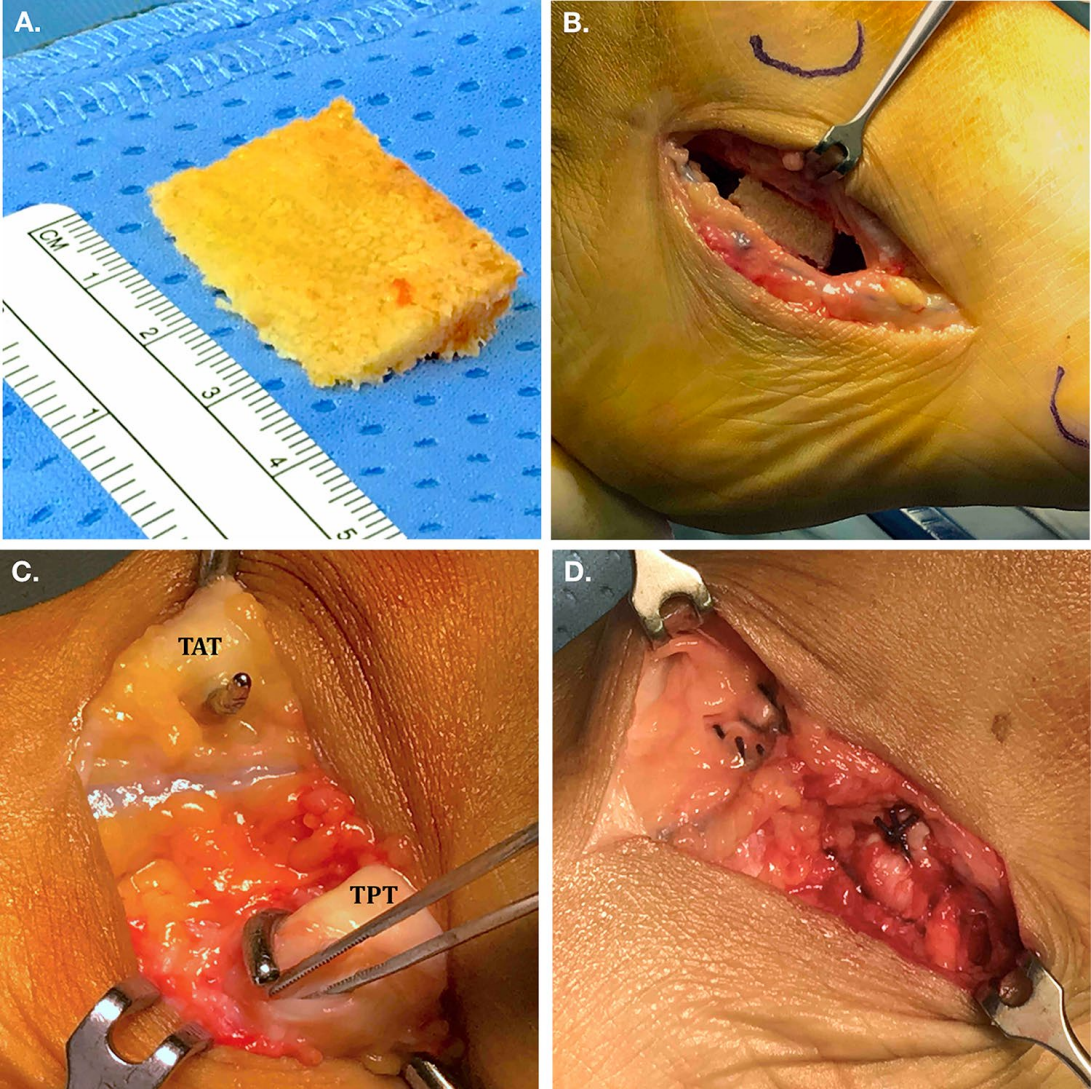
Intra-operative pictures of the main surgical steps. The preparation of allograft bone wedge (**a**) and the final lateral aspect of the calcaneus after open wedge osteotomy with allograft augmentation (**b**). The identification of the tibialis anterior tendon (TAT) and tibialis posterior tendon (TPT) (**c**) and final intraoperative aspect of TAT and TPT tenodesis (**d**)

Femoral head allograft (weight, 70 to 100 g) was first modeled to obtain a bony wedge (Fig. 3). The height of the bony wedge was calculated on radiographic planning on the TCA (Saltzman view [12]): a correction of 3° corresponded to 4 mm wedge height, 6° to 8 mm and 9° to 12 mm [15].

The calcaneus osteotomy was performed with lateral oblique incision to the calcaneus. The sural nerve was isolated and protected. A periosteal flap was elevated and the calcaneus osteotomy with an angle of 45° from longitudinal axis of the foot was then completed. A Dwyer osteotomy was used to create the lateral open wedge [16].

The allograft bony wedge was then inserted into the calcaneal space and fixed with percutaneous plantar 2 mm K-wire.

**Fig. 4:**
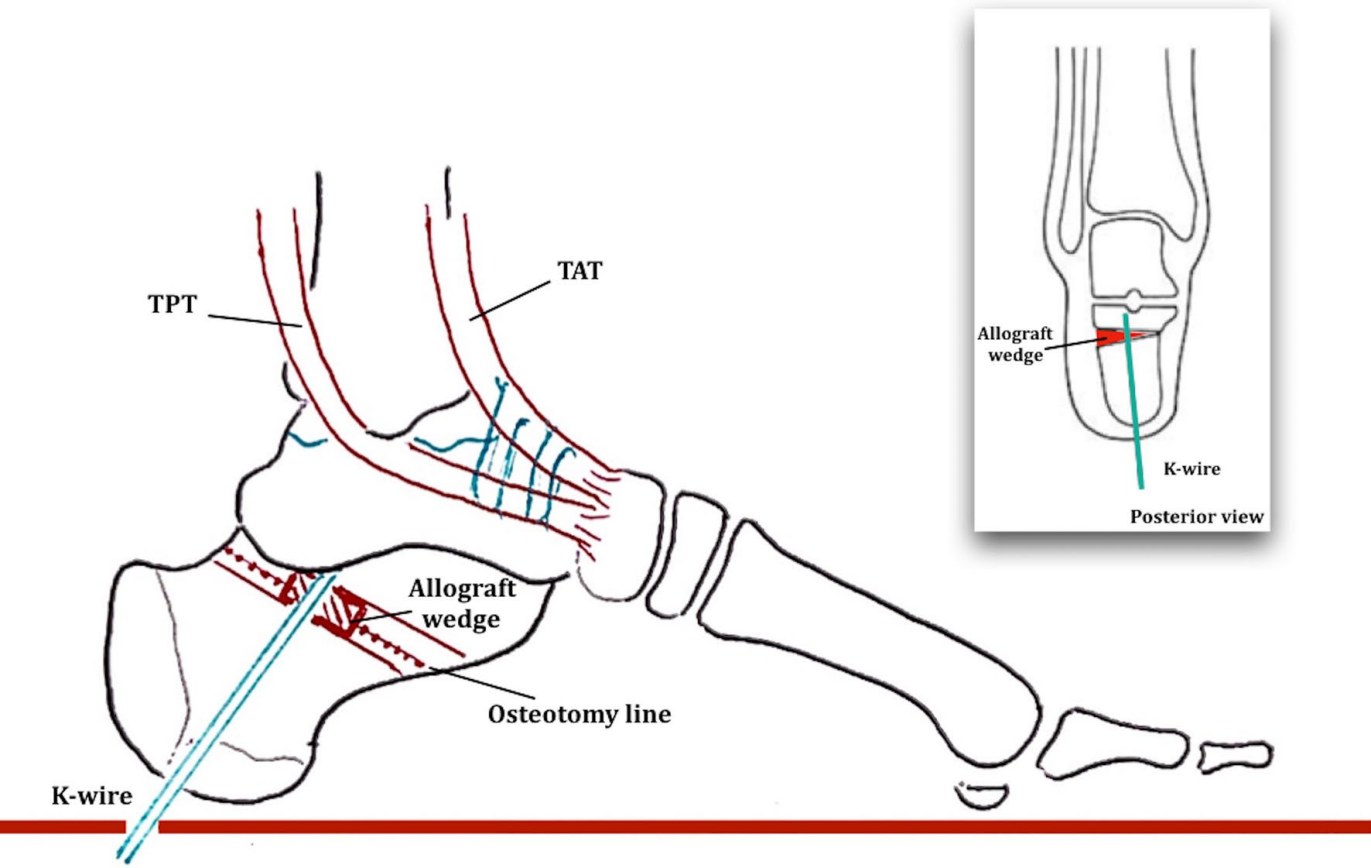
Schematic drawing of the surgical technique of lateral open wedge calcaneal osteotomy and bony allograft augmentation combined with tibialis anterior tendon (TAT) and tibialis posterior tendon (TPT) tendesis

A dorso-medial incision and a blunt dissection of the TPT and TAT were performed. A tenodesis between the two tendons was completed with no. 2 absorbable Vicryl suture. The schematic drawing of the operative technique is reported in Fig. 4.

The indications for TAT and TPT tenodesis were: insufficiency of the TPT not responding to conservative measures with local edema, pain in the navicular tendon insertion and/or along its course, confirmed by tendon inhomogeneity and tenosynovitis on high-resolution US and/or MRI. Patients with severely abnormal TPT or complete tear TPT were treated without tenodesis and excluded from the present analysis.

A 90° below-knee cast with nonweight-bearing for 4 weeks was positioned to stabilize the correction.

The X-ray examinations were performed on postoperative day 1 and 3 months after surgery. The K-wire was removed after 4 weeks and the patient was subsequently immobilized with a walking boot brace and partial weight-bearing for additional 4 weeks. After this period a progressive full weight-bearing was allowed.

Patients were clinically evaluated during outpatient consultations at 3, 6 and 12 months after surgery and on demand thereafter.

All patients were assessed with weight-bearing radiographs at the latest follow-up assessment.

## Statistical analysis

Continuous variables were reported as mean ± standard deviation (SD) and range of values and categorical variables were expressed as the absolute number and/or percentage.

A *post-hoc* calculation was performed considering the final AOFAS score as the primary outcome measure. With the AOFAS value of 48.4 ± 13.4 points reported in the literature for patients affected by AAFD [17] and the probability of type I error with α value of 0.01, the resulted *post-hoc* power of the present study on 22 patients was Φ (10.48) corresponding to 100% of power.

The Shapiro–Wilk test was performed to identify the normally distributed groups of values. The paired Student T-test was used to compare differences between normally distributed matched groups of data. The nonparametric Wilcoxon signed Rank test was used to compare not normally distributed matched groups of data. Statistical significance was set for *p* value < 0.01.

## Results

Fifty patients were identified as meeting the inclusion criteria. Twenty-two among them underwent combined talo-navicular arthrodesis, 3 were untraceable, 2 patients refused to participate at final clinical assessment and 1 had bilateral correction.

A total of 22 patients were retrospectively reviewed with a mean follow-up of 51.3 ± 35.9 (range, 24–122; 95% confidence interval [CI], 35.2–66.3) months.

All the included patients underwent lateral open wedge calcaneus osteotomy with allograft augmentation and TPT and TAT tenodesis.

The mean age at surgery was 59.8 ± 17.4 (range, 30–85; 95% CI: 52.1–67.5) years, 9 on 22 (40.9%) patients were female and 13 (59.1%) were male.

Fifteen procedures were performed on the left foot and 7 on the right foot.

All the included patients demonstrated a preoperative Myerson stage II.

The mean preoperative value of VAS score was 7.7 ± 0.7 (range, 7–9; 95% CI: 7.4–7.9) points, of AOFAS was 42.3 ± 10.1 (range, 18–68; 95% CI: 38.7–45.3) points, of FADI module was 38.3 ± 6.4 (range, 32–55; 95% CI: 35.5–41.2) points and of FAAM score was 36.6 ± 6.2 (range, 30–52; 95% CI: 33.8–39.3) points.

There was a significant improvement of the postoperative mean VAS (*p* < 0.001), AOFAS (*p* < 0.001), FADI (*p* < 0.001) and FAAM (*p* < 0.001) scores. The mean preoperative and final scores are reported in Table 1.

**Table 1:** Results of clinical outcome scores

	Preoperative value	LFU value	*p* value
Clinical score			
VAS	7.7 ± 0.7 (7–9)	1.8 ± 1.6 (0–7)	< 0.001
AOFAS	42.3 ± 10.1 (18–68)	85.7 ± 8.8 (69–100)	< 0.001
FADI	38.3 ± 6.4 (32–55)	85.3 ± 16.2 (30–104)	< 0.001
FAAM	36.6 ± 6.2 (30–52)	71.8 ± 11.2 (25–84)	< 0.001
Radiological angles			
TCA (°)	15.3 ± 2.4 (11–21)	5.1 ± 1.7 (0–9)	< 0.001
LTMT (°)	22.8 ± 5.7 (8–30)	10.4 ± 4.9 (3–20)	< 0.001
CP (°)	9.1 ± 2.9 (3–17)	21.3 ± 3.9 (11–32)	< 0.001

Continuous variables are listed with main, standard deviations and range of values (under parenthesis)

*CP* calcaneal pitch, *LFU* latest follow-up assessment, *LTMT* talo-first metatarsal angle, *TCA* talo-calcaneal angle

The subjective satisfaction evaluation showed that 19 out of 22 (86.4%) patients resulted very satisfied or satisfied for the clinical result, 2 partially satisfied and 1 unsatisfied.

All patients returned to regular activities of daily living.

One (4.5%) failure 7 years after surgery occurred with advanced degenerative changes of TN joint and persistent pain. The patient was treated with subsequent TN arthrodesis.

A CRPS was documented in 2 (9.1%) overweight and smoker patients, 3 months after surgery. No infection, wound leakage or allograft intolerance were reported.

There was a significant overall improvement of the TCA (*p* < 0.001) from 15.3 ± 2.4 (range, 11–21; 95% CI 13.6–15.2) degrees to 5.1 ± 1.7 (range, 0–9; 95% CI 4.2–5.8) degrees. The LMTM significantly (*p* < 0.001) changed from the preoperative value of 22.8 ± 5.7 (range, 8–30; 95% CI 18.8–22.1) degrees to the final value of 10.4 ± 4.9 (range, 3–20; 95% CI 9.1–11.8) degrees. The CP significantly (*p* < 0.001) improved from the preoperative value of 9.1 ± 2.9 (range, 3–7; 95% CI 6.9–8.6) degrees to the final value of 21.3 ± 3.9 (range, 11–32; 95% CI 18.5–21.6) degrees. Table 1 shows the preoperative and postoperative values of all treated patients.

## Discussion

The main findings of the present study were that the lateral open wedge calcaneal osteotomy with bony allograft augmentation and TPT and TAT tenodesis represents a successful procedure with good clinical and radiological results and high rate of patient’s satisfaction for AAFD at mid-term follow-up (Table 2).

**Table 2:** Details of preoperative active tibio-tarsal range of motion (ROM) of included patients

ROM analysis	Preoperative value	LFU value	*p* value
Plantarflexion (°)	39.8 ± 3.3	38.6 ± 4.3	0.305
Dorsiflexion (°)	28.1 ± 2.1	27.0 ± 2.6	0.130
Flexion extension total ROM (°)	67.6 ± 4.9	65.7 ± 5.5	0.233
Inversion (°)	26.8 ± 2.6	25.5 ± 2.9	0.125
Eversion (°)	16.9 ± 2.5	16.1 ± 1.2	0.183
Prono-supination total ROM (°)	43.4 ± 5.2	41.6 ± 4.1	0.209

Until now, few studies have been conducted on the wedge calcaneal osteotomies [11, 18, 19]. Nevertheless, the medial displacement calcaneal osteotomy (MDCO) is widely described combined with several soft tissue procedures [20] including flexor digitorum longus [17], flexor hallucis longus transfer [21] and lateral column lengthening [22].

Wacker et al. [23] reported 97.7% of excellent clinical outcomes and 81.8% of good alignment on a series of 44 patients treated with MDCO and flexor digitorum longus transfer at 4.3 years after surgery. Chadwick et al. [17] confirmed the same positive results at 15.2 years with 87% of patients completely satisfied for the surgery and 12.9% of failure rate, demonstrating that clinical results of this technique are durably maintained over time.

The clinical results of the present study are aligned with that reported in the literature with other operative techniques for AAFD [17, 24, 25] with 4.5% of failure rate and 86.4% of patient resulted satisfied with good clinical scores and radiologic correction at mid-term follow-up.

Some theoretical advantages of the described operative technique could be underlined: first the absence of long term calcaneal fixation device (i.e., screw or plate) and related comorbilities (intolerance, mobilization and rupture) [25]; second the large interface of cancellous bone tissue and stable mechanical support provided by the bony wedge could avoid the mismatch sometimes described for MDCO [20] providing high union rate of the osteotomy and good correction; third the degrees of “in varus” correction, like in other joints, can be precisely calculated with the preoperative plan [15].

The TN joint and the subtalar joint (STJ) are involved in pathogenesis AAFD and TN and STJ arthrodesis are both feasible options for the treatment of advanced AAFD [26] also in combination with the present surgical technique.

Although several tendon procedures [17, 18, 21, 22] are often associated to calcaneal osteotomy to sustain the medial structures of the foot, the TPT and TAT tenodesis [11] has been demonstrated comparable to other described techniques, without related comorbidities (i.e., donor-site deficit, extensor or flexor weakness)[27]. The involvement of TPT is thought to be one of the possible causes of pain in AAFD [28] and the described tenodesis is a possible technique to improve the function and pain of a damaged TPT.

Several limitations of the present research can be highlighted: this is a retrospective study conducted on limited number of patients with only mid-term follow-up. Furthermore, the main outcome measures are only based on clinical evaluation without soft tissue imaging techniques to precisely evaluate the possible failure of tenodesis.

Further studies are necessary to define the advantages of lateral open wedge calcaneal osteotomy and medial tendon procedures for the treatment of stage II of AAFD.

Nevertheless, the clinical results of the present research are satisfactory with low failure rate (4.5%) and high patients’ subjective satisfaction.

In conclusion lateral open wedge calcaneus osteotomy with allograft bony wedge combined with TAT and TPT tenodesis represents a feasible and reproducible surgical option for AAFD and demonstrated good mid-term results and low recurrence rate and complications.
